# Supplementary figures and images for: Characterization and complete genome sequence of Privateer, a highly prolate Proteus mirabilis podophage
Source: PeerJ. 2021 Feb 10;9:e10645. doi: 10.7717/peerj.10645 (PMC7881722; doi:10.7717/peerj.10645)

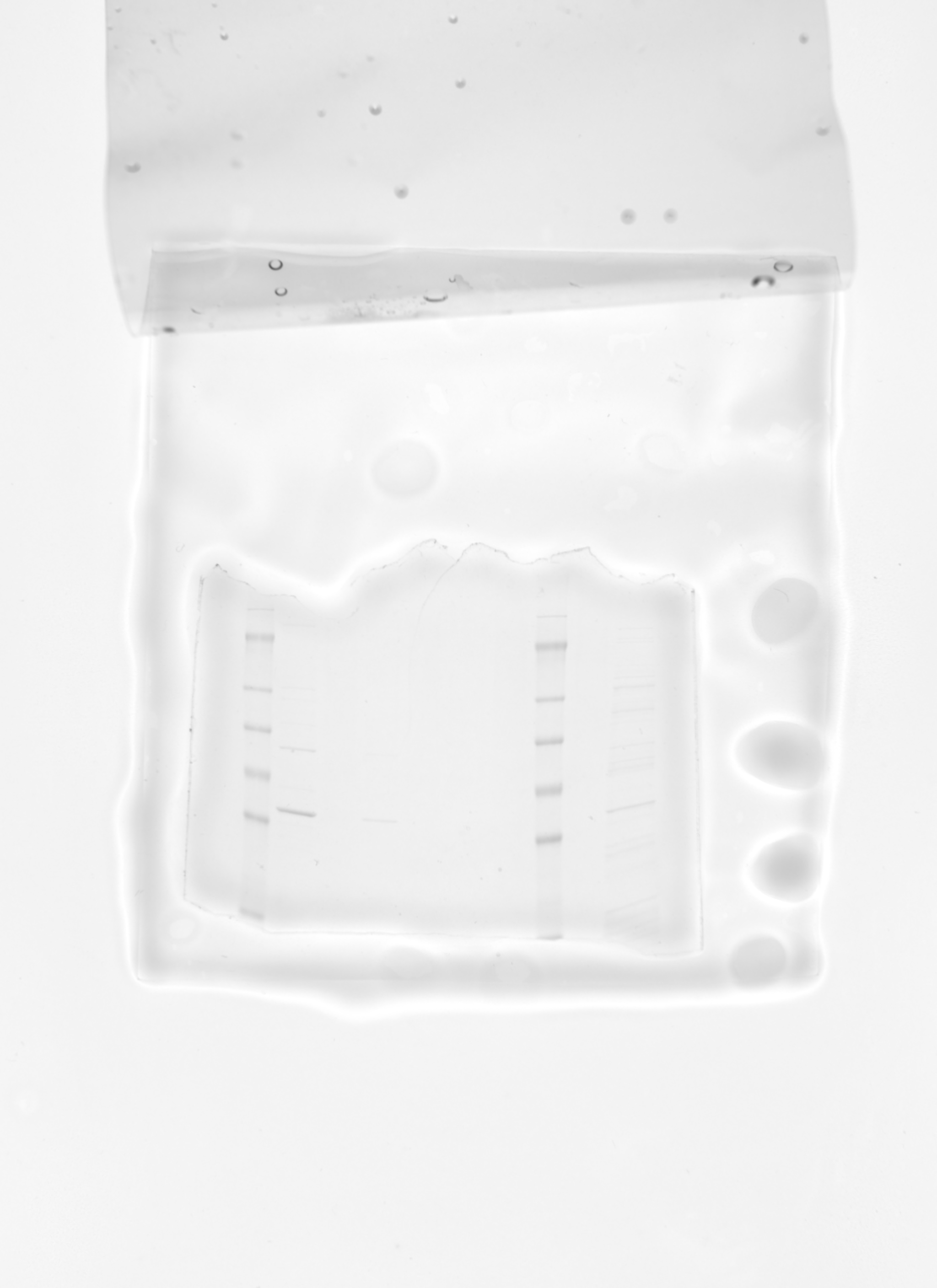

Supplement: Supplemental Information 2 — Original acquisition of SDS-PAGE shown in Fig. 5A. [file peerj-09-10645-s002.png]
